# Correction factors for the drag and pressure flows of power‐law fluids through rectangular ducts

**DOI:** 10.1002/pen.26344

**Published:** 2023-05-12

**Authors:** Christian Marschik, Wolfgang Roland

**Affiliations:** ^1^ Competence Center CHASE GmbH Linz Austria; ^2^ Institute of Polymer Processing and Digital Transformation Johannes Kepler University Linz Linz Austria

**Keywords:** modeling, processing, simulations

## Abstract

There are many industrial examples of low Reynolds number non‐Newtonian flows through rectangular ducts in polymer processing. They occur in all types of manufacturing processes in which raw polymeric materials are converted into products, ranging from screw extrusion to shaping operations in dies and molds. In addition, they are found in numerous rheological measurement systems. The literature provides various mathematical formulations for non‐Newtonian flows through rectangular ducts, but—if not simplified further—their solution usually requires use of numerical techniques. Removing the need for these time‐consuming techniques, we present novel analytical correction factors for the drag and pressure flows of power‐law fluids in rectangular flow channels. We approximated numerical results for a fully developed flow under isothermal conditions using symbolic regression based on genetic programming. The correction factors can be applied to the analytical theory that describes the flow of power‐law fluids between parallel plates to include effects of the side walls in the prediction of flow rate and viscous dissipation.

## Graphical abstract

Hybrid modeling approach including analytical, numerical, and data‐based modeling techniques.

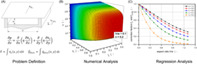

## 1 INTRODUCTION

The analysis of polymer‐melt flows in rectangular ducts has been the subject of many studies. Numerous flow situations of various physical complexities have been formulated mathematically and solved based on different solution methods. The most prominent examples can be found in a variety of textbooks.^[^ ^1^, ^2^, ^3^, ^4^ ^]^ For rectangular ducts, pure pressure flow was first addressed by Boussinesq,^[^ ^5^ ^]^ while the first mathematical model of a combined drag and pressure flow was published anonymously^[^ ^6^ ^]^ and later extended by Rowell and Finlayson^[^ ^7^ ^]^ and Maillefer.^[^ ^8^ ^]^ Assuming the flow of an incompressible Newtonian fluid in a straight rectangular channel, the analyses provided exact analytical solutions for the flow rate.

The complexity of the mathematical model increases when the shear‐thinning behavior of polymer melts is included, as the dependency of viscosity on shear rate leads to nonlinear differential equations. For rotationally symmetric geometries, such as pipes and annular gaps, the flow can be described by an ordinary differential equation with one independent variable. For rectangular ducts, in contrast, the problem cannot be reduced to one dimension without any further simplifications. Even for a purely viscous fluid, the mathematical problem involves a partial differential equation, solving which requires in most cases use of numerical methods. Middleman^[^ ^9^ ^]^ applied a finite difference scheme to independently solve the drag and pressure flows of a power‐law fluid in a rectangular duct. He further provided shape factors for selected processing conditions to demonstrate the influence of the side walls on the discharge rate. Later, Wheeler and Wissler^[^ ^10^ ^]^ and Palit and Fenner^[^ ^11^ ^]^ computed the combined drag and pressure flow for power‐law fluids based on the finite‐difference and finite‐element methods, respectively. To include polymers with Newtonian plateau at low shear rate, Sochi^[^ ^12^ ^]^ developed analytical solutions for the flow of Carreau and Cross fluids in thin slits, which require a numerical procedure to determine the shear rate at the channel wall.

Although recent progress in computer technology has pushed back computational barriers to numerically solving increasingly complex flows, the need remains for fast exact or approximate analytical solutions. Avoiding time‐consuming and computationally expensive numerical techniques, analytical equations significantly reduce calculation time and are therefore particularly useful in time‐critical applications such as practical design and optimization tasks. One approach to removing the need for numerical methods is to apply correction factors to the existing analytical theory available for basic geometries. Assuming the flow of a Newtonian fluid, Rauwendaal^[^ ^13^ ^]^ approximated the exact closed‐form analytical solution for a combined drag and pressure through a rectangular duct to derive shape factors for the drag and pressure flows in the form of linear functions:

(1) $$f_{d,R}=1-0.571\frac{h}{w},$$

(2) $$f_{p,R}=1-0.625\frac{h}{w}.$$

These expressions were developed for typical values of the aspect ratio of metering channels in single‐screw extruders (h/w<0.6). Focusing on the pressure flow of power‐law fluids through rectangular ducts, a few authors proposed correction factors to include wall effects in the prediction of the flow rate or pressure drop. Schenkel^[^ ^14^ ^]^ developed a set of analytical approximations for different aspect ratios. Similarly, Köpplmayr and Miethlinger^[^ ^15^ ^]^ introduced a correction factor using a second‐order polynomial function (Equation 3), whose coefficients depend on the power‐law index.

(3) $$f_{p,K}=a{\left(\frac{h}{w}\right)}^{2}+b\frac{h}{w}+c$$

In contrast, White and Huang^[^ ^16^ ^]^ (Equation 4) introduced generalized relationships for the correction factor that are continuous across their whole applications range. While the theories presented in Rauwendaal, Schenkel and White and Huang^[^ ^13^, ^14^, ^16^ ^]^ proposed corrections to the flow rate, Equation (3) was designed to adjust the pressure drop. Lang and Michaeli^[^ ^17^ ^]^ derived correction functions to include ducts of irregular cross section.

(4) $$f_{p,W}=\frac{1}{{\left(1+\frac{n^{1/3}\;3^{h/w}}{2\;w/h}\right)}^{1/n}}.$$

We have developed new analytical correction factors for the flow of a power‐law fluid in rectangular channels of finite width. Particular attention was paid to the calculation of the flow rate and viscous dissipation of a drag flow and a pressure flow. Numerical simulations were carried out for a fully developed flow under isothermal conditions. The primary aim was to approximate the difference between (i) numerical solutions obtained for the flow in finite channels and (ii) exact analytical results available for the flow in infinite channels by using symbolic regression based on genetic programming. The correction factors can be applied to account for the effects of the side walls in predicting the volume flow rate and the viscous dissipation of drag and pressure flows in rectangular ducts. These flows can be found in a variety of polymer‐processing machines including most prominently screw extrusion, dies, and molds. Avoiding numerical methods, the correction factors were designed to increase prediction accuracy in the analysis of drag and pressure flows in a broad range of equipment. Application fields range from design and optimization to troubleshooting tasks.

## 2 ANALYTICAL MODELING

### 2.1 Problem definition

In the first step, we derive the governing equations for the flows under investigation. Let us consider a straight rectangular duct of width w and height h with Cartesian coordinates oriented as shown in Figure 1. Two physical conditions are investigated: (i) a drag flow, where the upper plate moves in the down‐channel direction z at velocity $v_{b,z}$ and the pressure difference between channel inlet and outlet is zero, and (ii) a pressure flow with stationary boundaries governed by a down‐channel pressure gradient ∂p/∂z. In most polymer‐processing operations, the flow is governed either by a relative motion of one or more boundaries of the flow channel or by the presence of pressure gradients in the flow domain. In some applications, a combination of both flow components can be found such as in the metering zone of screw extruders. Assuming the flow of a Newtonian fluid, the discharge rate in this example results from a linear superposition of a drag and a pressure flow. The linear superposition, however, is invalid for shear‐thinning fluids, where the flow components are interrelated due to the dependency of viscosity on shear rate and the fluid velocities are more complex than the drag and pressure flow velocities profiles linearly superimposed. Complexity is further compounded by the combined effect of shear in all directions of the screw channel. The literature provides various numerical analyses of a combined drag and pressure flow of power‐law fluids between parallel plates. Examples include Rotem and Shinnar,^[^ ^18^ ^]^ Narkis and Ram,^[^ ^19^ ^]^ and Roland and Miethlinger.^[^ ^20^ ^]^ The validity of the linear superposition was examined by Kroesser and Middleman,^[^ ^21^ ^]^ who compared the relative errors between numerical solutions of the combined drag and pressure flow and those resulting from the superposition principle.

**FIGURE 1:**
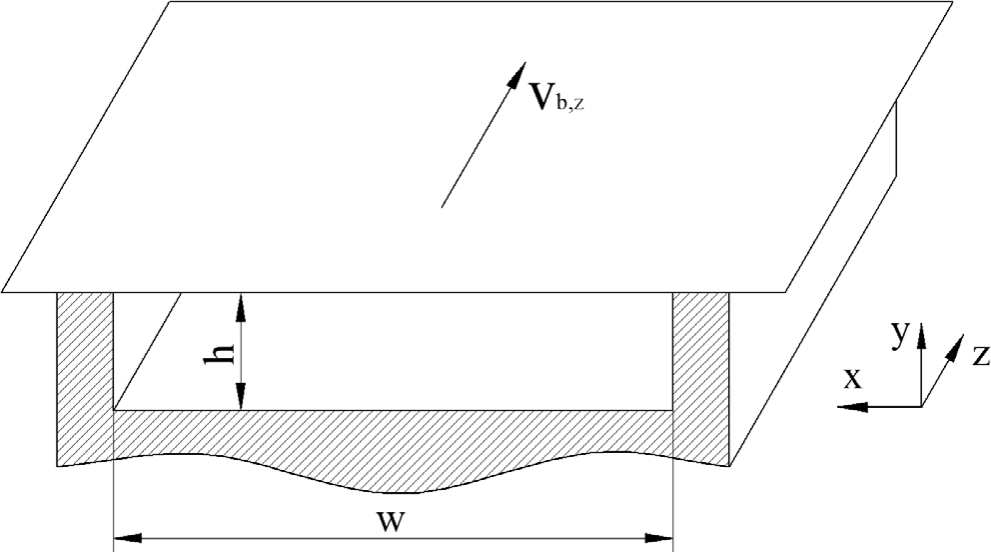
Rectangular flow channel of height h and width w.

We start the analysis by simplifying the conservation equations of mass, momentum, and energy. The following assumptions are made: (i) the flow is independent of time, fully developed, and isothermal, (ii) the fluid is incompressible and wall‐adhering, and (iii) gravitational and inertia forces are omitted. On the basis of these simplifications, the continuity and the energy equations vanish, and the problem is described by the momentum equation in the z direction, which reduces to a nonlinear partial differential equation with one nonzero velocity component $v_{z}=f\left(x,y\right)$:

(5) $$\frac{\partial p}{\partial z}=\frac{\partial \tau_{\mathrm{zx}}}{\partial x}+\frac{\partial \tau_{\mathrm{zy}}}{\partial y}.$$

Note that for pure drag flow, the down‐channel pressure gradient is zero (∂p/∂z=0) and the inhomogeneous part of the momentum equation vanishes. Considering a fully developed flow under isothermal conditions allows both the continuity and the energy equations to be omitted. To express the stress responses in the momentum equation, the polymer melt is treated as an inelastic viscous fluid described by the following generalized Newtonian‐fluid constitutive equation:

(6) τ=2ηD,

(7) $$D=\frac{1}{2}\left(L+L^{T}\right),\;L=\nabla v,$$

where the stress tensor τ is related to the rate‐of‐deformation tensor D obtained from the symmetric part of the velocity‐gradient tensor L. The shear‐thinning behavior of the polymer melt is modeled by a power law (Equation 8), where K is the consistency and n the power‐law exponent. The latter is a measure of the shear‐thinning behavior of the polymer melt; the lower the power‐law exponent, the more shear‐thinning the fluid.

(8) $$\eta =K\;{\left|\dot{\gamma }\right|}^{n-1}.$$

The magnitude of the shear rate is related to the second invariant of the rate‐of‐deformation tensor. For an incompressible fluid, it is calculated by:

(9) $$\left|\dot{\gamma }\right|=\sqrt{2\left(D:D\right)}.$$

With these definitions, the viscosity of the polymer melt is obtained from:

(10) $$\eta =K{\left[{\left(\frac{\partial v_{z}}{\partial x}\right)}^{2}+{\left(\frac{\partial v_{z}}{\partial y}\right)}^{2}\right]}^{\frac{n-1}{2}}.$$

The power law has been widely used to approximate the viscosity behavior of polymer melts in various flow analyses. A disadvantage of the model is that it is incapable of describing the Newtonian plateau at low shear rates, where it predicts an infinite value for the zero‐shear viscosity. This might be problematic for pressure flows, where the shear rate varies from a zero value on the symmetry axis to a maximum value at the wall. A more complex viscosity function that accurately approximates the rheological behavior of polymer melts in the terminal plateaus, the shear‐thinning regime, and the transitions between them is the Carreau–Yasuda model^[^ ^22^, ^23^ ^]^:

(11) $$\eta_{C}=\eta_{\infty }+\left(\eta_{0}-\eta_{\infty }\right){\left(1+{\left(\lambda \;\dot{\gamma }\right)}^{a}\right)}^{\frac{n_{C}-1}{a}},$$

where $\eta_{0}$ and $\eta_{\infty }$ are the zero‐shear and infinite‐shear viscosities, respectively, λ is the characteristic relaxation time, and $n_{C}$ the Carreau–Yasuda power‐law index. The parameter a defines the width of the transition between the Newtonian plateau and the shear‐thinning region. To combine the simplicity of the power law and the accuracy of the Carreau–Yasuda equation, the model parameters of the latter can be transformed into equivalent power‐law parameters. On a log–log scale, the power law is a linear function and can be considered as local tangent to the Carreau–Yasuda model at any given shear rate, which may be obtained from:

(12) $${\dot{\gamma }}_{\mathrm{eff}}=\frac{v_{\mathrm{ref}}}{h}=\frac{\dot{V}}{w\;h^{2}}.$$

The local power‐law parameters result from the slope and the intercept of the tangent:

(13) $$n=1+\frac{\left(\eta_{0}-\eta_{\infty }\right)\left(n_{C}-1\right){\left(\lambda \;{\dot{\gamma }}_{\mathrm{eff}}\right)}^{a}{\left(1+{\left(\lambda \;{\dot{\gamma }}_{\mathrm{eff}}\right)}^{a}\right)}^{\frac{n_{C}-1-a}{a}}\;}{\eta_{\infty }+\left(\eta_{0}-\eta_{\infty }\right){\left(1+{\left(\lambda \;{\dot{\gamma }}_{\mathrm{eff}}\right)}^{a}\right)}^{\frac{n_{C}-1}{a}}}.\;$$

(14) $$K=\eta_{\infty }+\left(\eta_{0}-\eta_{\infty }\right){\left(1+{\left(\lambda \;{\dot{\gamma }}_{\mathrm{eff}}\right)}^{a}\right)}^{\frac{n_{C}-1}{a}}{{\dot{\gamma }}_{\mathrm{eff}}}^{1-n}.$$

Finally, the momentum equation is written as:

(15) $$\frac{\partial p}{\partial z}=\frac{\partial }{\partial x}\left(\eta \frac{\partial v_{z}}{\partial x}\right)+\frac{\partial }{\partial y}\left(\eta \frac{\partial v_{z}}{\partial y}\right).$$

Solving Equation (15) in combination with the corresponding boundary conditions in Table 1 yields the down‐channel velocity profiles of drag and pressure flows, whose volume flow rates result from:

(16) $$\dot{V}=\int v_{z}\left(x,y\right)\;\mathrm{dA}.$$

**TABLE 1:** Velocity boundary conditions.

Coordinates		Drag flow	Pressure flow
x	y	$v_{z}$	$v_{z}$
0	y	0	0
w	y	0	0
x	0	0	0
x	h	$v_{b,z}$	0

The total viscous dissipation rate per unit down‐channel length is obtained by integrating the specific dissipation rate over the cross‐sectional area, which is part of the energy equation (${\dot{q}}_{\text{diss}}=\tau :L$):

(17) $${\dot{Q}}_{\text{diss}}=\int {\dot{q}}_{\text{diss}}\left(x,y\right)\;\mathrm{dA}.$$

(18) $${\dot{q}}_{\text{diss}}=\eta \left[{\left(\frac{\partial v_{z}}{\partial x}\right)}^{2}+{\left(\frac{\partial v_{z}}{\partial y}\right)}^{2}\right].$$

### 2.2 Theory of similarity

For convenience, the governing flow equations are transformed into a dimensionless form by using the theory of similarity. To this end, a set of dimensionless variables is introduced:

(19) $$\;\xi =\frac{x}{w},\;\psi =\frac{y}{h},\;v_{z}=\frac{v_{z}}{v_{\mathrm{ref}}}.$$

Traditionally, two dimensionless systems have been used, in which the reference velocities are defined as:

(20) $$v_{\mathrm{ref},1}=v_{b,z}=v_{z}\;\cos\left(\varphi \right).$$

(21) $$v_{\mathrm{ref},2}=\bar{v}=\frac{\dot{V}}{w\;h}.$$

The first (Equation 20) is commonly applied in the analysis of combined drag and pressure flows in unrolled metering channels of single‐screw extruders and describes the down‐channel velocity of the moving barrel surface.^[^ ^24^ ^]^ The second (Equation 21) is typically employed in the modeling of pure pressure flows with stationary boundaries as found in dies, molds, or melt‐filtration systems.^[^ ^25^ ^]^ In this analysis, we use both definitions of the reference velocity to derive two dimensionless formulations of the nonlinear boundary value problem. While the correction factors are then developed based on the reference system of a combined drag and pressure flow, we additionally illustrate how the results can be transformed to the reference system of a pure pressure flow. The viscosity in Equation (10) is rewritten to:

(22) $$\eta^{*}=\frac{\eta \;h^{n-1}}{K\;v_{\mathrm{ref}}^{n-1}}={\left[{\left(\frac{h}{w}\right)}^{2}{\left(\frac{\partial v_{z}}{\partial \xi }\right)}^{2}+{\left(\frac{\partial v_{z}}{\partial \;\psi }\right)}^{2}\right]}^{\frac{n-1}{2}}$$

The momentum equations to be solved for each reference system are then:

(23) $$6\Pi_{p,z}={\left(\frac{h}{w}\right)}^{2}\;\frac{\partial }{\partial \xi }\left(\eta^{*}\frac{\partial v_{z}}{\partial \xi }\right)+\frac{\partial }{\partial \psi }\left(\;\eta^{*}\frac{\partial v_{z}}{\partial \psi }\right).\;$$

(24) $${\hat{\Pi }}_{p,z}={\left(\frac{h}{w}\right)}^{2}\;\frac{\partial }{\partial \xi }\left(\eta^{*}\frac{\partial v_{z}}{\partial \xi }\right)+\frac{\partial }{\partial \psi }\left(\;\eta^{*}\frac{\partial v_{z}}{\partial \psi }\right).\;$$

where the dimensionless pressure gradients are defined by:

(25) $$\Pi_{p,z}=\frac{p_{z}^{\prime }\;h^{n+1}}{6\;K\;v_{b,z}^{n}}$$

(26) $${\hat{\Pi }}_{p,z}=\frac{\left|p_{z}^{\prime }\right|\;h^{n+1}}{K\;{\bar{v}}^{n}}$$

Finally, flow and dissipation rates are expressed in dimensionless form:

(27) $$\Pi_{V}=\frac{2\;\dot{V}}{w\;h\;v_{b,z}}=2\int_{0}^{1}\int_{0}^{1}v_{z}\left(\xi ,\psi \right)\;\mathrm{d\xi}\;\mathrm{d\psi}$$

(28) $${\hat{\Pi }}_{V}=\frac{\dot{V}}{w\;h\;\bar{v}}=1=\int_{0}^{1}\int_{0}^{1}v_{z}\left(\xi ,\psi \right)\;\mathrm{d\xi}\;\mathrm{d\psi}$$

(29) $$\Pi_{Q}=\frac{{\dot{Q}}_{\text{diss}}\;h^{n}}{w\;K\;v_{\mathrm{ref}}^{n+1}\;}=\int_{0}^{1}\int_{0}^{1}\pi_{Q}\left(\xi ,\psi \right)\;\mathrm{d\xi}\;\mathrm{d\psi}$$

with:

(30) $$\pi_{Q}={\left[{\left(\frac{h}{w}\right)}^{2}{\left(\frac{\partial v_{z}}{\partial \xi }\right)}^{2}+{\left(\frac{\partial v_{z}}{\partial \psi }\right)}^{2}\right]}^{\frac{n+1}{2}}.$$

Table 2 summarizes the dimensionless boundary conditions of the nonlinear partial differential Equations (23) and (24). While exact analytical solutions are available for a Newtonian fluid with n=1, ^[^ ^2^ ^]^ shear‐thinning fluids with n<1 give rise to a nonlinear boundary value problem, whose solution requires use of numerical methods. The results presented in this work were obtained by using the finite‐volume method.

**TABLE 2:** Dimensionless velocity boundary conditions.

Coordinates		Drag flow	Pressure flow
ξ	ψ	$v_{z}$	$v_{z}$
0	ψ	0	0
1	ψ	0	0
ξ	0	0	0
ξ	1	1	0

### 2.3 Correction factors

To consider the effects of the side walls on flow and dissipation rates, we derived correction factors for the non‐Newtonian flows. To this end, we used the dimensionless model of a combined drag and pressure flow (reference system 1), in which the reference velocity is defined by the velocity of the moving barrel surface in Equation (20). The numerical results were related to exact analytical solutions available for the simplified case in which the channel is infinitely wide. For shallow channels with h/w<0.1, ^[^ ^3^ ^]^ the flow can be assumed to occur in the y–z mid plane. In this simplified model, the dependency of the down‐channel velocity $v_{z}$ on the cross‐channel coordinate x can be ignored and the problem reduces to a nonlinear ordinary differential equation. Physically, this means that the side‐edge effects are omitted. Under these conditions, the dimensionless flow and dissipation rates as defined in Equations (27) and (29) are equal to one in the case of a drag flow:

(31) $$\Pi_{V,d}=1,\;\Pi_{Q,d}=1$$

For a pressure flow in the first reference system, in contrast, the target variables are obtained from the following relationships^[^ ^26^ ^]^:

(32) $$\Pi_{V,p}=-\mathrm{sign}\left(\Pi_{p,z}\right)\frac{3^{\frac{1}{n}}\;n}{2n+1}{\left|\Pi_{p,z}\right|}^{\frac{1}{n}}$$

(33) $$\Pi_{Q,p}=\frac{3^{\frac{n+1}{n}}\;n}{2n+1}{\left|\Pi_{p,z}\right|}^{\frac{n+1}{n}}$$

The correction factors were defined as the ratios of the numerical results obtained for the rectangular duct and the corresponding exact analytical solutions for the flow between parallel plates:

(34) $$f_{d}=\frac{\Pi_{V,\mathrm{sim}}}{\Pi_{V,d}},\;f_{d,\text{diss}}=\frac{\Pi_{Q,\mathrm{sim}}}{\Pi_{Q,d}}.$$

(35) $$f_{p}=\frac{\Pi_{V,\mathrm{sim}}}{\Pi_{V,p}\;},\;f_{p,\text{diss}}=\frac{\Pi_{Q,\mathrm{sim}}}{\;\Pi_{Q,p}}.\;$$

In pressure flows viscous dissipation rate is equal to the pumping power $P=\dot{V}∆p$. In the dimensionless reference system of a combined drag and pressure flow, dissipation rate is therefore obtained from:^[^ ^26^ ^]^

(36) $$\Pi_{Q,\mathrm{sim}}=3\;\Pi_{V,\mathrm{sim}}\;\Pi_{p,z}=3\;f_{p}\;\Pi_{V,p}\;\Pi_{p,z}=f_{p}\;\Pi_{Q,p.}$$

which demonstrates that $f_{p}=f_{p,\text{diss}}$. Note that the correction factor defined in Equation (35) can only be applied to combined drag and pressure flow. This is due to the definition of the dimensionless down‐channel velocity in Equation (20), which uses the down‐channel velocity of the moving barrel surface as reference velocity. To enable the use of the correction factor in the case of pure pressure flow with stationary boundaries, we combined Equations ((25), (26), (27), (28)) and (32) to derive the following transformation equation:

(37) $${\hat{\Pi }}_{p,z}=\frac{2^{n+1}{\left(2n+1\right)}^{n}}{n^{n}}\;\frac{1}{f_{p}^{n}}$$

When using the mean fluid velocity as reference velocity (Equation 21), the dimensionless flow rate is constant with ${\hat{\Pi }}_{V}=1$ (Equation 28). Rather than correcting the flow rate, the second reference system hence requires the dimensionless pressure gradient to be corrected.

### 2.4 Parametric study

The nonlinear boundary value problem defined in Section 2.2 has two dimensionless input parameters: (i) the power‐law exponent n and (ii) the channel aspect ratio h/w. In the case of a drag flow, the dimensionless down‐channel pressure gradient $\Pi_{p,z}$ is zero, while in the case of a pressure flow dimensionless analysis shows that $\Pi_{V}\sim \Pi_{p,z}^{1/n}$ and $f_{p}\neq f\left(\Pi_{p,z}\right)$, as illustrated in Equation 32. In other words, the same results are obtained for all other values of the dimensionless down‐channel pressure gradient.

In the next step, n and h/w were varied to create a set of 126 physically independent modeling setups. The ranges of variation are shown in Table 3. The power‐law exponent, for example, was varied between 0.2 and 1.0, which includes most polymer melts in industrial use. Moreover, the aspect ratio was varied between 0 and 1.3, which represents a broad range of equipment. In screw extrusion, for example, the aspect ratio of conventional metering channels is in the range of 0<h/w<0.15. The screw channel, however, may become boxier when more advanced screw sections with multiple flights are considered. These are commonly part of high‐performance screws such as barrier or wave‐dispersion screws, where the aspect ratio of the channel can exceed h/w>1.0. Similar geometrical boundaries can be found in extrusion dies or molds. Depending on the shape of the product, different aspect ratios are possible. Examples include flat films with h/w<0.05 and profiles with h/w>1.0.

**TABLE 3:** Range of values of h/w and n.

Quantity	Minimum	Maximum	Increment
h/w	0	1.3	0.1
n	0.2	1.0	0.1

Note that the volume flow and dissipation rates of an infinitely wide channel (h/w=0) are obtained from Equations (32) and (33), respectively. For Newtonian fluids with n=1, exact analytical solutions are used to calculate the flow and dissipation rates for channels of finite width.^[^ ^2^ ^]^

## 3 NUMERICAL MODELING

Omitting modeling setups with n=1 and h/w=0, numerical solutions for the dimensionless volume flow rate $\Pi_{V}$ and dimensionless dissipation rate $\Pi_{Q}$ at the remaining 104 physically independent design points were calculated by using the finite volume method implemented in the software package ANSYS Academic Fluent.^[^ ^27^ ^]^ For each combination of dimensionless influencing parameters, we generated and solved a dimensional representation of the flow equations and translated the solutions back into dimensionless space. A fast computational solving process was enabled by an automatically driven parameterized setup in terms of geometry, material properties, and operating conditions. The simulation approach was initially validated by comparing numerical results to exact closed‐form analytical solutions for the Newtonian case. For details on the numerical solving process, see Roland et al. and Marschik et al.^[^ ^24^, ^28^ ^]^

### 3.1 Numerical solutions

Figure 2 plots the correction factors for the flow rate (a) and the dissipation rate (b) of a pure drag flow ($\Pi_{p,z}=0$) as functions of the channel aspect ratio for various power‐law exponents. By definition, $f_{d}$ and $\;f_{d,\text{diss}}$ reach unity for h/w=0, which constitutes an isothermal flow between parallel plates. The influences of the side walls on flow and dissipation rates become more pronounced the higher the aspect ratio of the channel. While the former decreases with increasing aspect ratio, the latter shows the opposite behavior. This result is directly related to the down‐channel velocity profile of the polymer melt, which approaches zero close to the walls due to wall adhesion.

**FIGURE 2:**
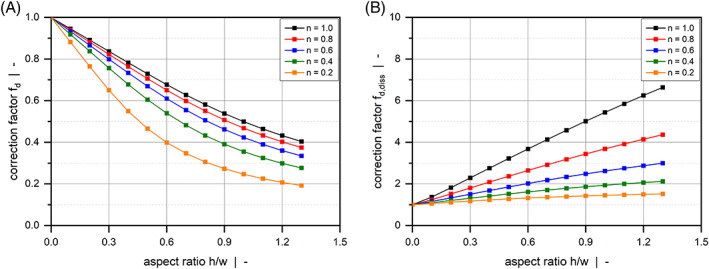
Drag flow of a power‐law fluid through a rectangular duct. Numerical solutions for $f_{d}$ and $f_{d,\text{diss}}$ for various channel aspect ratios and power‐law indices.

Figure 3 and Figure 4 illustrate contour plots of the velocity distribution of a wide channel (Figure 3) and a narrow channel (Figure 4) for various power‐law indices. When the width is large compared to the height (h/w=0.1), the channel produces the widely known linear velocity profile over large parts of the width with $v_{z}\left(\xi ,0\right)=0$ and $v_{z}\left(\xi ,1\right)=1$. For all power‐law exponents, the velocity field is only marginally affected by the side walls. The significance of the wall effects is increased when a square duct (h/w=1.0) is considered, where a nonlinear velocity distribution can be observed even for a Newtonian fluid. Two effects are observed: First, rather than being restricted to the regions close to the side walls, the velocity gradients in ξ‐direction extend to the center of the channel, thereby affecting the entire velocity distribution. Second, the velocities approach zero over a pronounced region in the lower half of the cross section, which in turn leads to increased velocity gradients in ψ‐direction close to the moving plate. In the case of a drag flow, side‐wall effects lower the discharge rate and increase dissipation. For all aspect ratios, wall effects become more pronounced the more shear‐thinning the fluid.

**FIGURE 3:**
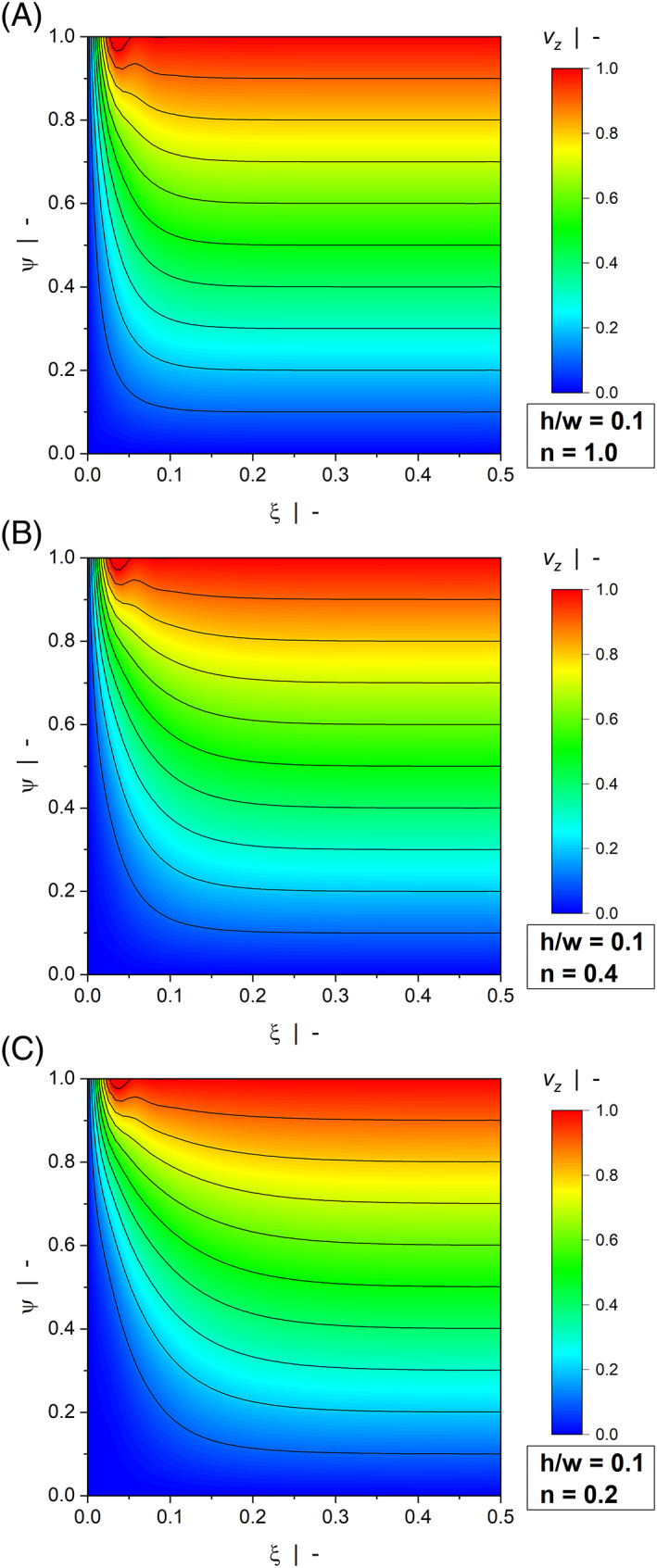
Contour plots of down‐channel velocity profiles $v_{z}\left(\xi ,\psi \right)$ of a drag flow for h/w=0.1: n=1.0 (A), n=0.4 (B), and n=0.2 (C).

**FIGURE 4:**
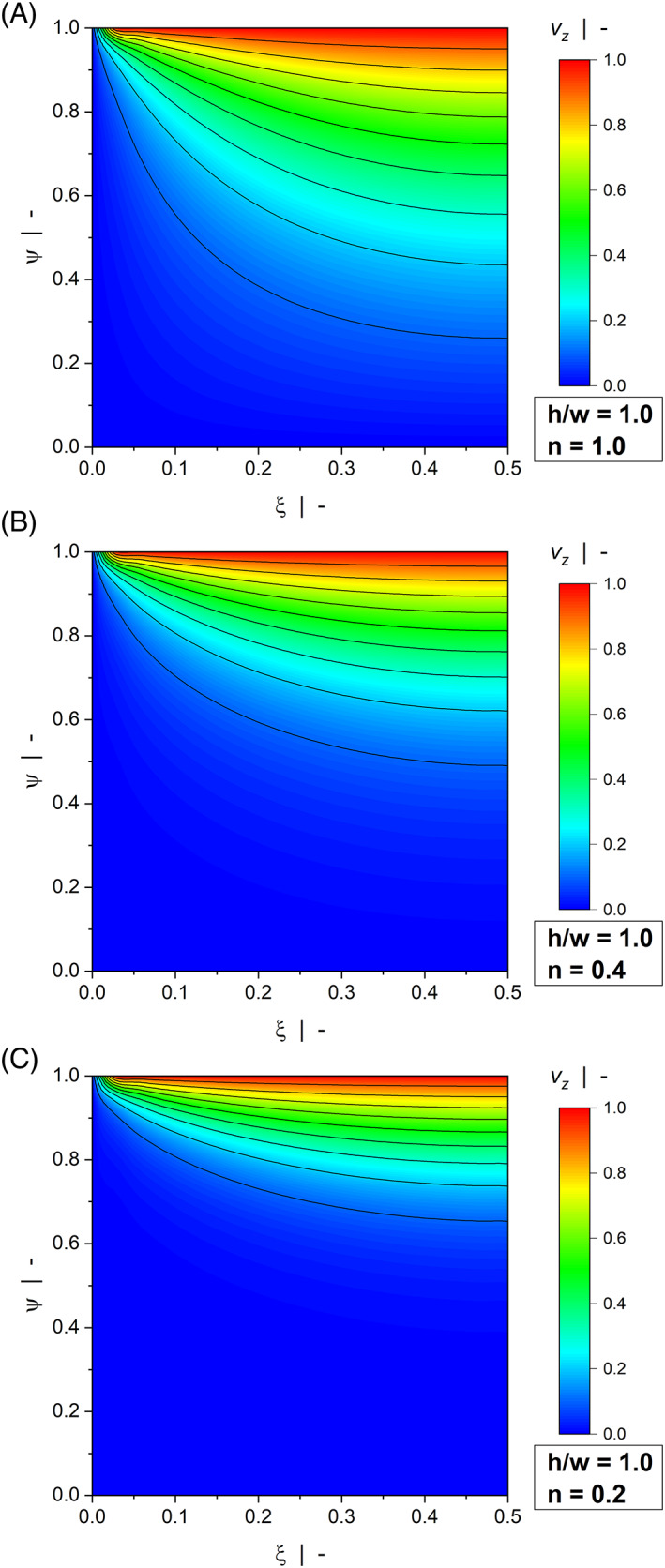
Contour plots of down‐channel velocity profiles $v_{z}\left(\xi ,\psi \right)$ of a drag flow for h/w=1.0: n=1.0 (A), n=0.4 (B), and n=0.2 (C).

Figure 5 shows the correction factors for the flow rate and the dissipation rate of a pure pressure flow as functions of the channel aspect ratio for various power‐law exponents. As in the previous example, the rate‐limiting effect of the side walls increases with increasing aspect ratio. However, for all power‐law indices the flow‐rate reduction is stronger than in the case of a drag flow. Furthermore, as $f_{p}=f_{p,\text{diss}}$, both flow and dissipation rates decrease with increasing aspect ratio.

**FIGURE 5:**
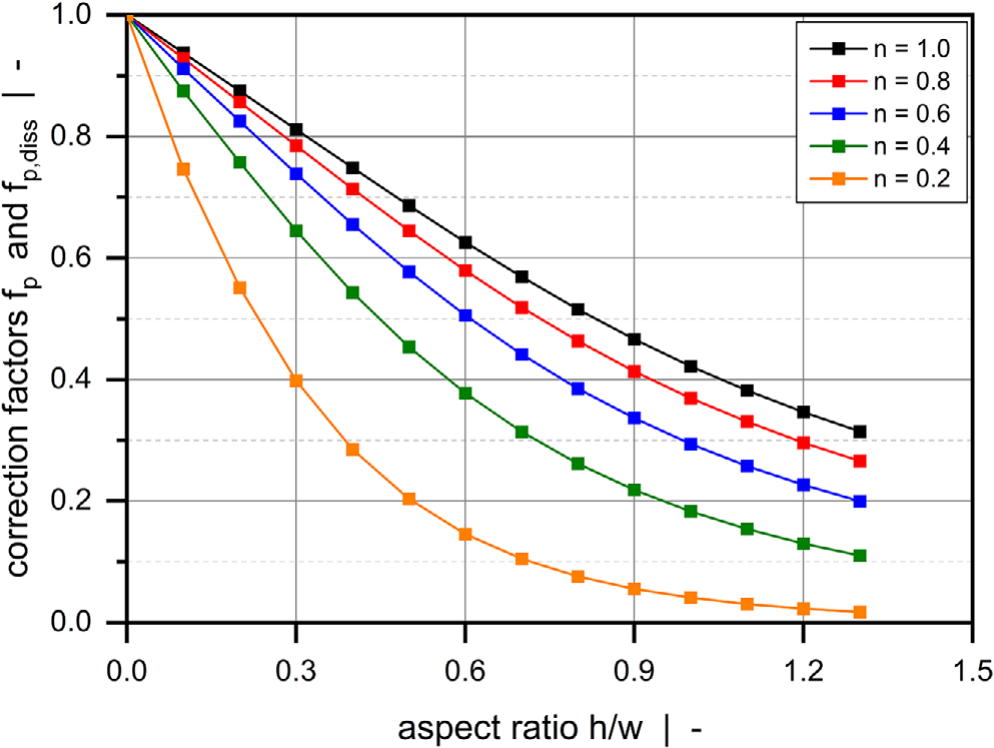
Pressure flow of a power‐law fluid through a rectangular duct. Solutions for $f_{p}=f_{p,\text{diss}}$ for various channel aspect ratios and power‐law indices.

Figures 6 and 7 show contour plots of the down‐channel velocity profiles of a wide channel (Figure 6) and a narrow channel (Figure 7). While the maximum velocity in the case of a drag flow is $v_{\max}=1$, the velocity magnitude in the case of a pressure flow is not restricted. To allow a qualitative comparison of the contour plots, we scaled the numerical solutions using $v/v_{\max}$, where $v_{\max}$ is the maximum velocity in each flow situation.

**FIGURE 6:**
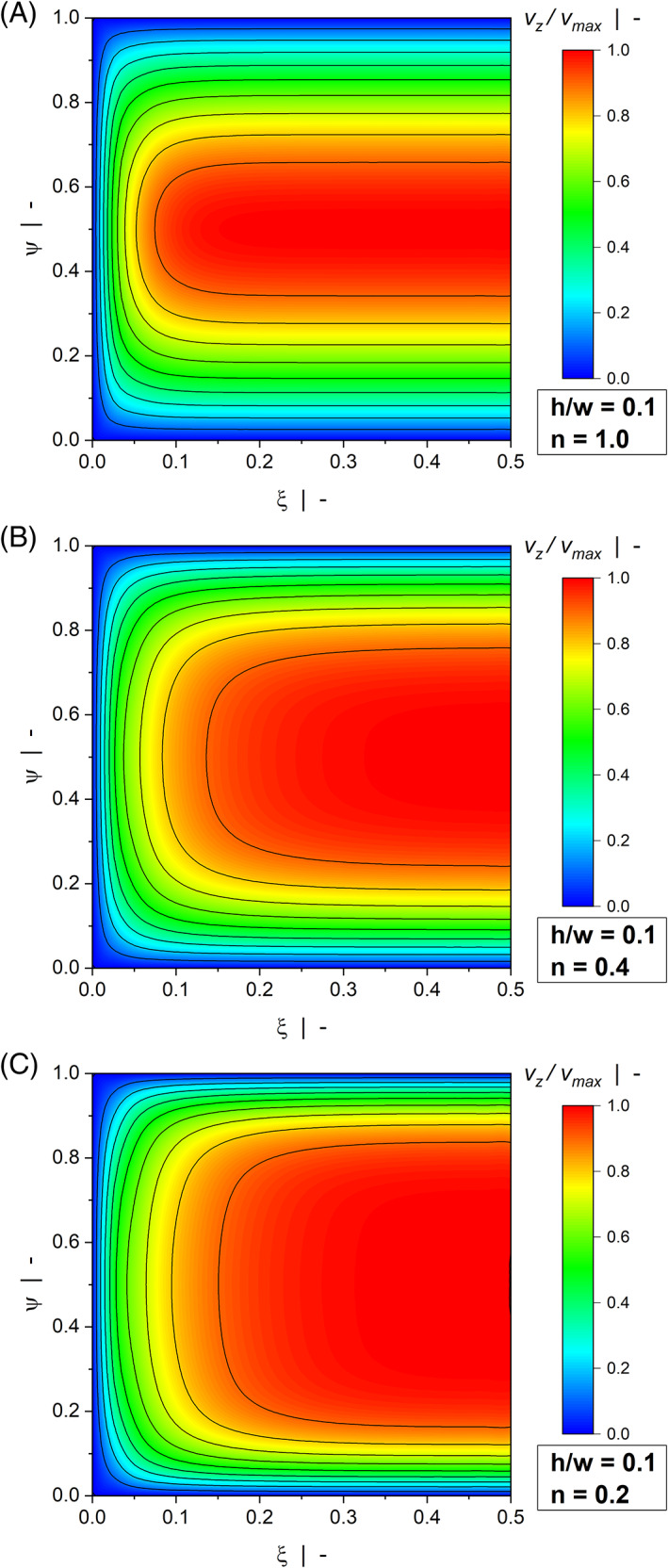
Contour plots of down‐channel velocity profiles $v_{z}\left(\xi ,\psi \right)/v_{max}$ of a pressure flow for h/w=0.1: n=1.0 (A), n=0.4 (B), and n=0.2 (C).

**FIGURE 7:**
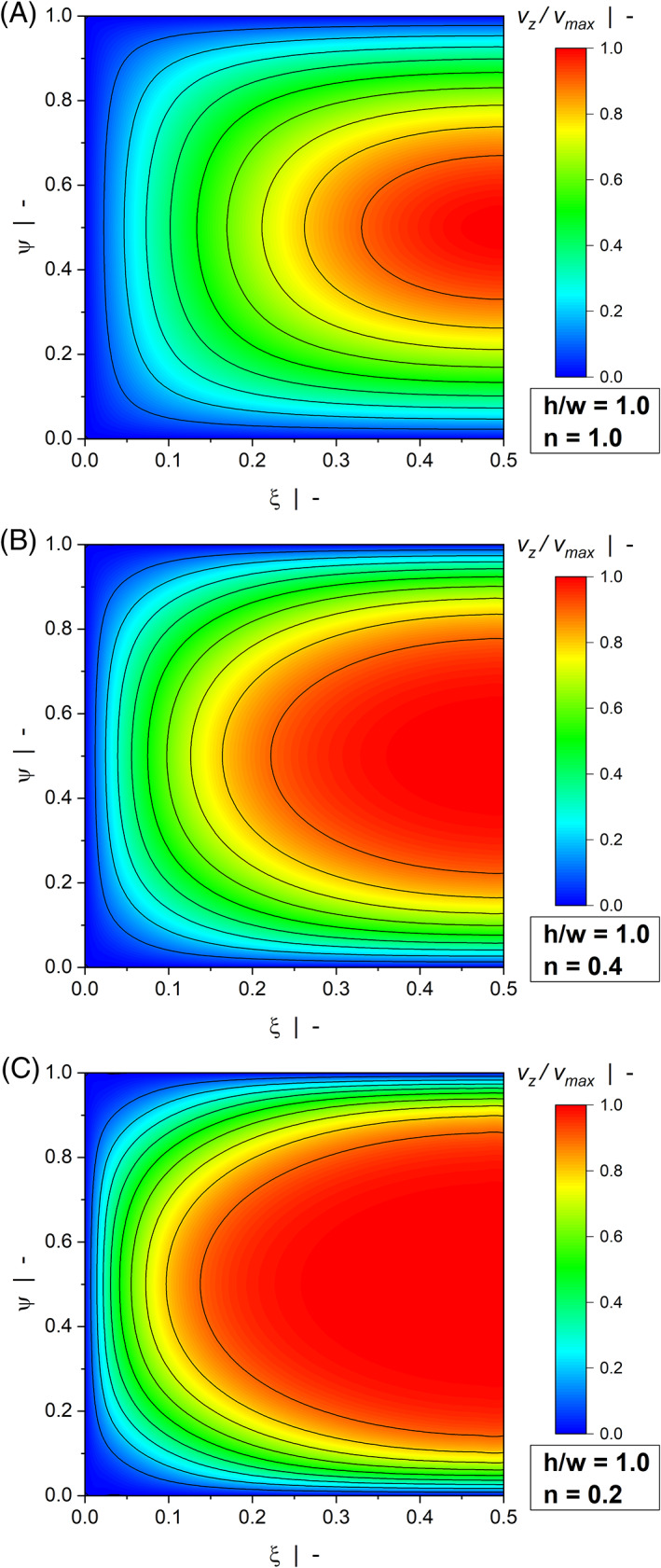
Contour plots of down‐channel velocity profiles $v_{z}\left(\xi ,\psi \right)/v_{max}$ of a pressure flow for h/w=1.0: n=1.0 (A), n=0.4 (B), and n=0.2 (C).

For a Newtonian fluid, the shallow channel (h/w=0.1) produces the well‐known parabolic velocity profile over large parts of the width. The maximum velocity is found in the channel center, while the velocity at the upper and lower walls is zero due to wall adhesion. In contrast to Figure 3, the contour plots show pronounced differences even for h/w=0.1. These are caused by the shear‐thinning flow behavior of the polymer melt. With decreasing power‐law exponent, the velocity gradients in ψ‐direction move to the regions close to the upper and lower walls, converting the distribution to a plug‐flow‐type profile. In addition, side wall effects become more pronounced, as indicated by the velocity gradients in ξ‐direction. This is particularly true when a square duct (h/w=1.0) is considered, where the side walls affect the entire velocity distribution. To demonstrate the significance of the side wall effects, Figure 8 shows the velocity profiles in the center of the channel $v_{z}\left(0.5,\psi \right)$ for a highly shear‐thinning fluid with n=0.2 and various aspect ratios. The plots compare our numerical results for the flow through a rectangular channel with the exact analytical solutions for the simplified model, in which the flow takes place between parallel plates and side wall effects are omitted (see Hopmann and Michaeli^[^ ^3^ ^]^). While the analytical solution is independent of the aspect ratio, the side walls reduce the velocity magnitudes in the numerical analysis, which in turn affect the flow and dissipation rates. The velocity differences between the shallow channel and the square duct correspond well to the behavior of the correction factor $f_{p}$ in Figure 5.

**FIGURE 8:**
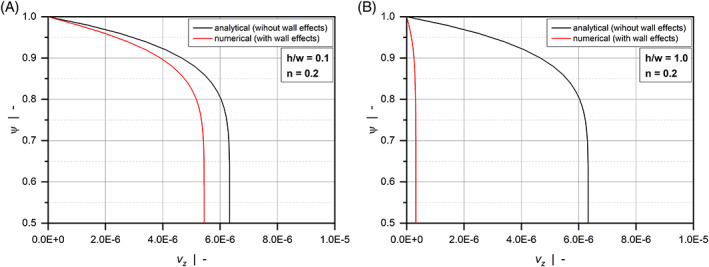
Dimensionless down‐channel velocity profiles $v_{z}\left(0.5,\psi \right)$ in the center of the cannel for n=0.2: h/w=0.1 (A) and h/w=1.0 (B). Comparison of the exact analytical solution for the flow between parallel plates and the corresponding numerical result with wall effects.

## 4 REGRESSION ANALYSIS

In the final step, we approximated our numerical results for the correction factors analytically, which are shown in Table A1 in the Appendix, using symbolic regression based on genetic programming. The objective was to develop relationships for $f_{d}$, $f_{d,\text{diss}}$, and $f_{p}$ as functions of the influencing parameters h/w and n. In contrast to classical regression methods, symbolic regression produces models in the form of mathematical expressions without pre‐defining a specific model structure. To restrict the search space for candidate models, we included only basic arithmetic operations as addition, subtraction, and multiplication in the function set. For regression analysis, we applied the offspring selection genetic algorithm (OSGA) implemented in the open‐source software HeuristicLab.^[^ ^29^ ^]^ This method optimizes model quality without taking into account model complexity. Model optimization was driven by a constant optimization evaluator which calculates Pearson's $R^{2}$ of a candidate solution according to Equation (41) and optimizes the constants used. For detailed information on symbolic regression, see Roland et al.^[^ ^30^ ^]^ The usefulness of this type of regression analysis has been demonstrated in the context of various polymer‐processing problems.^[^ ^24^, ^25^, ^26^, ^28^, ^31^, ^32^, ^33^, ^34^ ^]^

The data set of design points was divided into: (i) a training set and (ii) a test set, consisting of 81 and 45 random design points, respectively. For each correction factor, we performed 20 runs to generate a set of regression solutions, whose prediction accuracy were evaluated by means of the training and test sets. Our regression analysis provided the following analytical relationships for the correction factors:

(38) $$f_{d}=1-\frac{a_{0}\frac{h}{w}\left(\;a_{1}+\frac{h}{w}+a_{2}n\right)}{a_{3}+a_{4}\frac{h}{w}+{\left(\frac{h}{w}\right)}^{2}+a_{5}n+a_{6}\frac{h}{w}n}$$

(39) $$f_{d,\text{diss}}=1+\frac{b_{0}{\left(\frac{h}{w}\right)}^{2}\left(b_{1}+\frac{h}{w}+b_{2}n\right)\left(b_{3}+n\right)\;}{\left(\frac{h}{w}+b_{4}n\right)\;\left(b_{5}+n\right)}$$

(40) $$f_{p}=1+\frac{c_{0}\frac{h}{w}}{1+c_{1}\frac{h}{w}+c_{2}n+c_{3}\frac{1+c_{4}\frac{h}{w}+c_{5}n}{1+c_{6}\frac{h}{w}+\frac{c_{7}}{n}+c_{8}n}}$$

where the model coefficients are shown in Table A2 in the Appendix. To illustrate the accuracy of the equations, we calculated the volume flow rates and dissipations according to Equations (34) and (35) for all setups and compared the results to our numerical solutions. Table 4 summarizes the coefficients of determination, $R^{2}$ (Equation 41), the mean and the maximum absolute errors, ${\mathrm{AE}}_{\text{mean}}$ (Equation 42) and ${\mathrm{AE}}_{\max}$ (Equation 43), and the mean relative error ${\mathrm{RE}}_{\text{mean}}$ (Equation 44) for all 126 data points, which confirm the outstanding accuracy of the approximations. These were calculated by:

(41) $$R^{2}=1-\frac{\sum_{i=1}^{n}{\left(y_{i}-{\hat{y}}_{i}\right)}^{2}}{\sum_{i=1}^{n}{\left(y_{i}-\bar{y}\right)}^{2}}.\;$$

(42) $${\mathrm{AE}}_{\text{mean}}=\frac{1}{N}\sum_{i=1}^{n}\left|y_{i}-{\hat{y}}_{i}\right|$$

(43) $${\mathrm{AE}}_{\max}=\max\;\left(\left|y_{i}-{\hat{y}}_{i}\right|\right).$$

(44) $${\mathrm{RE}}_{\text{mean}}=\frac{1}{N}\sum_{i=1}^{n}\frac{\left|y_{i}-{\hat{y}}_{i}\right|}{y_{i}}.$$

where $y_{i}$ and ${\hat{y}}_{i}$ are the numerical and approximated results, respectively, and $\bar{y}$ is the mean of the numerical solutions. All models achieve a coefficient of determination of $R^{2}>0.999$, which demonstrates high prediction accuracy. In addition, Figure 9 and Figure 10 represent scatter plots for all correction factors, comparing numerical and approximated results for all sample points. Note that the correction factors shown in Equations ((38), (39), (40)) can be further simplified. For each value of the power‐law index, the expressions can be approximated by polynomial functions. A similar approach was presented by Köpplmayr and Miethlinger.^[^ ^15^ ^]^

**TABLE 4:** Quality measures for $f_{d}$, $f_{d,\text{diss}}$, and $f_{p}$.

Quality measure	Unit	$f_{d}$	$f_{d,\text{diss}}$	$f_{p}$
$R^{2}$	–	0.999971	0.999960	0.999976
${\mathrm{AE}}_{\text{mean}}$	–	0.000943	0.005865	0.001076
${\mathrm{AE}}_{\max}$	–	0.003406	0.028304	0.003962
${\mathrm{RE}}_{\text{mean}}$	%	0.21	0.30	0.65

**FIGURE 9:**
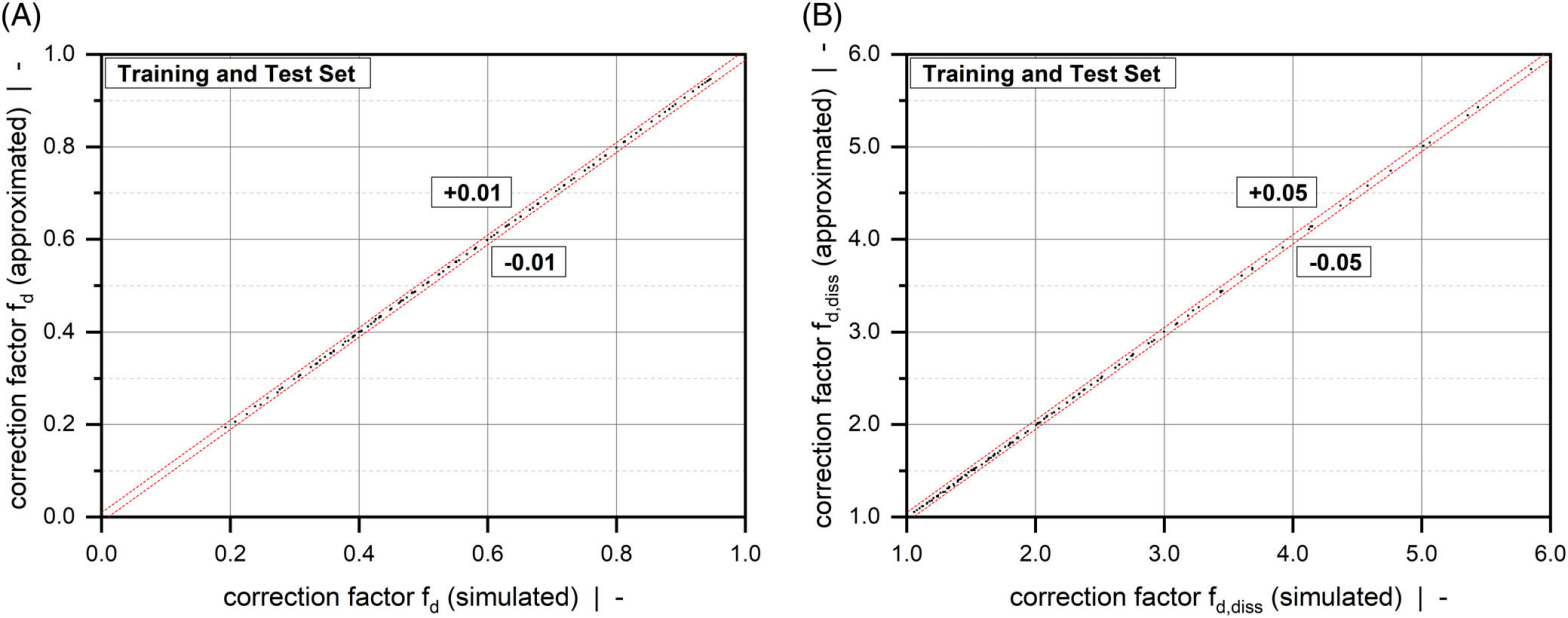
Scatter plots of $f_{d}$ (A) and $f_{d,\text{diss}}$ (B): Comparison between numerical and analytical solutions according to Equations (38) and (39). The dashed lines indicate absolute errors.

**FIGURE 10:**
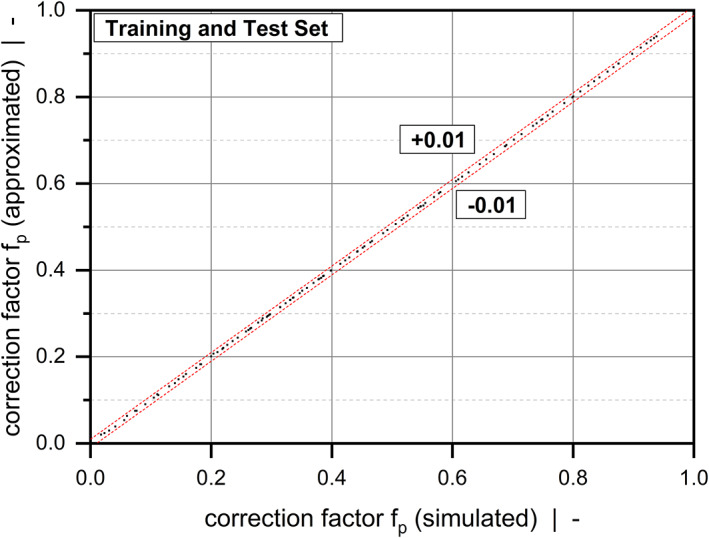
Scatter plot of $f_{p}$: Comparison between numerical and analytical solutions according to Equation (40). The dashed lines indicate absolute errors.

Finally, we compared the accuracy of our analytical relationship for $f_{p}$ in predicting the simulated results in the range of 0≤h/w≤1.0 with the solutions presented by Rauwendaal (Equation 2), Köpplmayr and Miethlinger (Equation 3), and White and Huang (Equation 4). To allow a quantitative comparison of the relationships, we transformed the correction factor proposed by Köpplmayr and Miethlinger on the basis of Equation (37). This step was required to account for the different approach taken by Köpplmayr and Miethlinger. Rather than correcting the flow rate, the authors developed a correction to the pressure gradient. Figure 11 and Table 5 illustrate the increased prediction accuracy of the new parameter.

(45) $$f_{p}=f_{p,K}^{1/n}.$$

**FIGURE 11:**
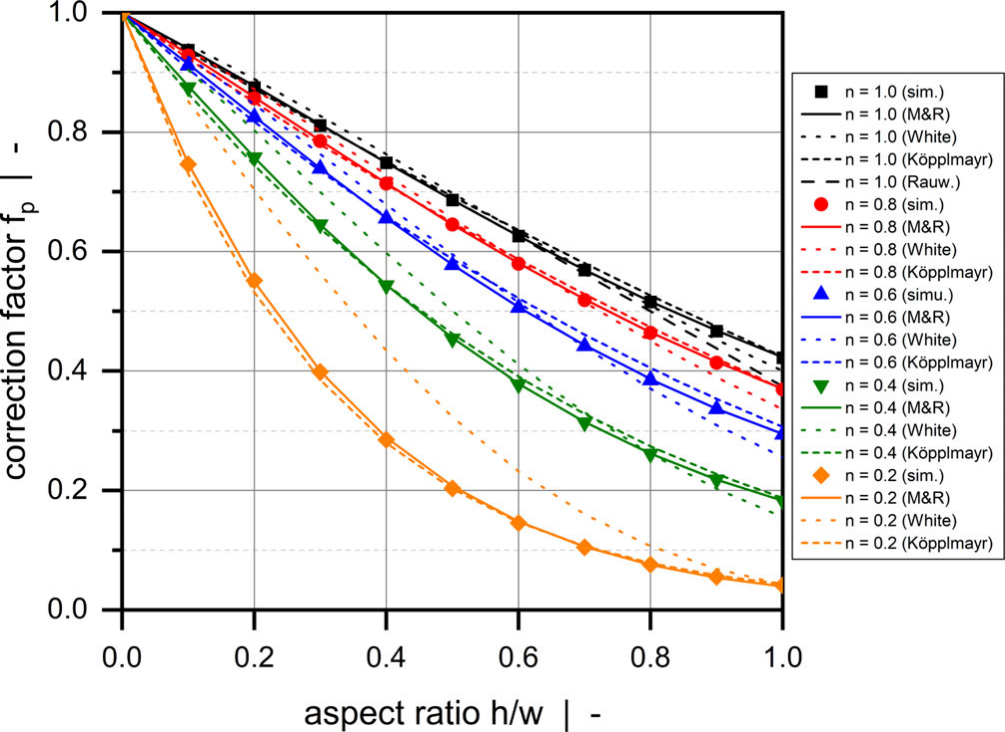
Comparison between numerical results and analytical approximations for $f_{p}$ according to Equations (2), (3), (4), and (40).

**TABLE 5:** Comparison of prediction accuracies: our $f_{p}$ versus $f_{p,K}$ and $f_{p,W}$.

Quality measure	Unit	$f_{p}$, new	$f_{p,K}$, equation (3)	$f_{p,W}$, equation (4)
$R^{2}$	–	0.999966	0.998644	0.966814
${\mathrm{AE}}_{\text{mean}}$	–	0.001139	0.007327	0.029969
${\mathrm{AE}}_{\max}$	–	0.003962	0.020462	0.163920
${\mathrm{RE}}_{\text{mean}}$	%	0.34	1.78	8.687

## 5 CONCLUSION

The analytical correction factors developed in this work can be used to consider the effects of the side walls in predicting the volume flow rate and the viscous dissipation of drag and pressure flows in rectangular ducts. They were derived from a large number of numerical results for a fully developed flow of an incompressible power‐law fluid under isothermal conditions and designed to correct exact analytical solutions available for the simplified case in which the polymer melt is conveyed between two parallel plates.^[^ ^3^ ^]^ Without resorting to numerical methods, the proposed relationships, whose applicability is restricted to ducts with 0≤h/w≤1.3 and polymer melts with 0.2≤n≤1.0, allow fast computation of realistic results for the actual flow situation. Note that while pressure flows through ducts with h/w>1.0 can be modeled by taking the inverse of the correction factors, similar assumption is invalid for drag flows.

A critical aspect in the application of the correction factors for the viscous dissipation rate is the isothermal assumption applied in the mathematical derivation, which restricts the validity of the theory in practical use. A convenient method that enables the use of the correction factors in non‐isothermal analyses is based on so‐called lumped‐parameter models. This approach, which has been applied in the modeling of extruder screws^[^ ^35^, ^36^ ^]^ and dies,^[^ ^15^ ^]^ divides the channel into short segments, within which the temperature is assumed to be uniform, thereby allowing the use of isothermal models. Rather than resolving spatial parameter variations over the channel cross section, a lumped form of the energy equation is solved to calculate the temperature change along the channel. For each segment, thermodynamic and rheological material properties are evaluated based on a mean cross‐sectional temperature. The correction factors presented here can be employed to include the influence of the side walls in the computation of local flow and dissipation rates. The use of the correction factors, however, is not limited to straight flow channels with constant cross sections; when employed in combination with network theory, they can also be applied to model multidimensional flows in geometries with changing cross sections. In this case, the flow domain is discretized into both down‐ and cross‐channel segments. The usefulness of network theory in the flow analysis of extruders and dies has been demonstrated in several studies.^[^ ^3^, ^37^, ^38^, ^39^ ^]^

## NOMENCLATURE

a
: Carreau–Yasuda parameter
$a_{\mathrm{ij}}$
: model coefficients of $f_{d}$
A
: cross‐sectional area
${\mathrm{AE}}_{\max}$
: maximum absolute error
${\mathrm{AE}}_{\text{mean}}$
: mean absolute error
$b_{\mathrm{ij}}$
: model coefficients of $f_{d,\text{diss}}$
$c_{\mathrm{ij}}$
: model coefficients of $f_{p}$
D
: rate‐of‐deformation tensor
$f_{d}$
: drag flow correction factor for flow rate
$f_{d,R}$
: drag flow correction factor for flow rate (Rauwendaal)
$f_{d,\text{diss}}$
: drag flow correction factor for dissipation rate
$f_{p}$
: pressure flow correction factor for flow rate
$f_{p,K}$
: pressure flow correction factor for flow rate (Köpplmayr and Miethlinger)
$f_{p,R}$
: pressure flow correction factor for flow rate (Rauwendaal)
$f_{p,W}$
: pressure flow correction factor for flow rate (White and Huang)
$f_{p,\text{diss}}$
: correction factor for dissipation rate (pressure flow)
h
: channel depth
K
: consistency
n
: power‐law exponent
$n_{C}$
: Carreau–Yasuda power‐law exponent
p
: pressure
$p_{z}^{\prime }$
: pressure gradient in z‐direction
${\dot{q}}_{\text{diss}}$
: dissipation rate per unit volume
${\dot{Q}}_{\text{diss}}$
: dissipation rate per unit length
$R^{2}$
: coefficient of correlation
${\mathrm{RE}}_{\text{mean}}$
: mean relative error
$v_{i}$
: velocities
$v_{b,z}$
: down‐channel plate velocity
$v_{\mathrm{ref}}$
: reference velocity
$\bar{v}$
: mean velocity
v
: velocity vector
$\dot{V}$
: volume flow rate
w
: channel width
x
: cross‐channel coordinate
y
: up‐channel coordinate
$y_{i}$
: numerical result
${\hat{y}}_{i}$
: approximated solution
$\bar{y}$
: mean value of numerical results
z
: down‐channel coordinate
$\dot{\gamma }$
: shear rate
${\dot{\gamma }}_{\mathrm{eff}}$
: effective shear rate
η
: viscosity
$\eta_{C}$
: viscosity (Carreau–Yasuda model)
$\eta_{0}$
: zero shear viscosity
$\eta_{\infty }$
: infinite shear viscosity
$\eta^{*}$
: dimensionless viscosity
$v_{i}$
: dimensionless velocities
λ
: characteristic relaxation time
ξ
: dimensionless cross‐channel direction
$\Pi_{p,z}$
: dimensionless pressure gradient in down‐channel direction (system 1)
${\hat{\Pi }}_{p,z}$
: dimensionless pressure gradient in down‐channel direction (system 2)
$\pi_{Q}$
: dimensionless specific dissipation rate
$\Pi_{Q}$
: dimensionless dissipation rate
$\Pi_{Q,\mathrm{sim}}$
: dimensionless dissipation rate (simulated)
$\Pi_{Q,d}$
: dimensionless dissipation rate (drag flow)
$\Pi_{Q,p}$
: dimensionless dissipation rate (pressure flow)
$\Pi_{V}$
: dimensionless volume flow rate (system 1)
${\hat{\Pi }}_{V}$
: dimensionless volume flow rate (system 2)
$\Pi_{V,\mathrm{sim}}$
: dimensionless volume flow rate (simulated)
$\Pi_{V,d}$
: dimensionless drag flow rate
$\Pi_{V,p}$
: dimensionless pressure flow rate
$\tau_{\mathrm{ij}}$
: stresses
τ
: stress tensor
*φ*
: screw pitch angle;
ψ
: dimensionless up‐channel direction

## Data Availability

Data available on request from the authors.

## APPENDIX A

**TABLE A1:** Numerical solutions for $f_{d}$, $f_{d,\text{diss}}$, and $f_{p}$ for all sample points.

n	h/w	$f_{d}$	$f_{d,\text{diss}}$	$f_{p}$	n	h/w	$f_{d}$	$f_{d,\text{diss}}$	$f_{p}$
1	0.1	0.946	1.369	0.938	0.5	0.7	0.524	1.921	0.386
0.9	0.1	0.944	1.297	0.934	0.4	0.7	0.482	1.707	0.314
0.8	0.1	0.941	1.240	0.928	0.3	0.7	0.426	1.525	0.220
0.7	0.1	0.938	1.193	0.921	0.2	0.7	0.347	1.362	0.105
0.6	0.1	0.933	1.156	0.912	1	0.8	0.581	4.579	0.516
0.5	0.1	0.927	1.125	0.898	0.9	0.8	0.567	3.791	0.492
0.4	0.1	0.919	1.099	0.875	0.8	0.8	0.551	3.184	0.463
0.3	0.1	0.905	1.077	0.835	0.7	0.8	0.531	2.710	0.429
0.2	0.1	0.882	1.057	0.747	0.6	0.8	0.506	2.336	0.385
1	0.2	0.891	1.822	0.875	0.5	0.8	0.474	2.036	0.331
0.9	0.2	0.887	1.652	0.867	0.4	0.8	0.433	1.790	0.262
0.8	0.2	0.882	1.519	0.857	0.3	0.8	0.378	1.581	0.175
0.7	0.2	0.875	1.413	0.843	0.2	0.8	0.306	1.396	0.076
0.6	0.2	0.866	1.330	0.825	1	0.9	0.538	5.012	0.467
0.5	0.2	0.854	1.262	0.799	0.9	0.9	0.524	4.124	0.442
0.4	0.2	0.837	1.206	0.757	0.8	0.9	0.507	3.438	0.414
0.3	0.2	0.811	1.159	0.687	0.7	0.9	0.487	2.903	0.379
0.2	0.2	0.764	1.118	0.552	0.6	0.9	0.462	2.482	0.336
1	0.3	0.837	2.289	0.812	0.5	0.9	0.431	2.143	0.284
0.9	0.3	0.831	2.016	0.800	0.4	0.9	0.391	1.866	0.218
0.8	0.3	0.823	1.804	0.785	0.3	0.9	0.339	1.633	0.140
0.7	0.3	0.813	1.638	0.766	0.2	0.9	0.273	1.426	0.056
0.6	0.3	0.799	1.506	0.739	1	1	0.499	5.435	0.422
0.5	0.3	0.782	1.400	0.702	0.9	1	0.485	4.446	0.398
0.4	0.3	0.757	1.314	0.645	0.8	1	0.468	3.683	0.370
0.3	0.3	0.718	1.241	0.555	0.7	1	0.448	3.089	0.335
0.2	0.3	0.651	1.177	0.399	0.6	1	0.424	2.620	0.294
1	0.4	0.783	2.757	0.749	0.5	1	0.393	2.245	0.244
0.9	0.4	0.774	2.381	0.733	0.4	1	0.355	1.938	0.183
0.8	0.4	0.764	2.089	0.714	0.3	1	0.307	1.680	0.112
0.7	0.4	0.751	1.862	0.689	0.2	1	0.247	1.453	0.041
0.6	0.4	0.733	1.682	0.656	1	1.1	0.464	5.848	0.382
0.5	0.4	0.711	1.538	0.610	0.9	1.1	0.450	4.759	0.358
0.4	0.4	0.678	1.420	0.543	0.8	1.1	0.433	3.920	0.330
0.3	0.4	0.630	1.321	0.442	0.7	1.1	0.414	3.266	0.297
0.2	0.4	0.550	1.232	0.285	0.6	1.1	0.390	2.752	0.258
1	0.5	0.729	3.222	0.687	0.5	1.1	0.361	2.340	0.210
0.9	0.5	0.719	2.743	0.668	0.4	1.1	0.325	2.004	0.154
0.8	0.5	0.706	2.372	0.645	0.3	1.1	0.280	1.723	0.091
0.7	0.5	0.690	2.083	0.616	0.2	1.1	0.225	1.478	0.031
0.6	0.5	0.670	1.855	0.577	1	1.2	0.432	6.249	0.346
0.5	0.5	0.643	1.671	0.526	0.9	1.2	0.418	5.062	0.323
0.4	0.5	0.605	1.522	0.454	0.8	1.2	0.402	4.148	0.296
0.3	0.5	0.551	1.395	0.351	0.7	1.2	0.383	3.437	0.264
0.2	0.5	0.466	1.281	0.204	0.6	1.2	0.360	2.877	0.226
1	0.6	0.677	3.682	0.626	0.5	1.2	0.333	2.430	0.182
0.9	0.6	0.665	3.100	0.605	0.4	1.2	0.299	2.067	0.130
0.8	0.6	0.651	2.650	0.580	0.3	1.2	0.258	1.764	0.074
0.7	0.6	0.633	2.299	0.547	0.2	1.2	0.207	1.500	0.023
0.6	0.6	0.610	2.022	0.506	1	1.3	0.404	6.641	0.315
0.5	0.6	0.580	1.800	0.451	0.9	1.3	0.390	5.356	0.292
0.4	0.6	0.540	1.618	0.378	0.8	1.3	0.375	4.368	0.266
0.3	0.6	0.483	1.463	0.278	0.7	1.3	0.356	3.600	0.235
0.2	0.6	0.399	1.324	0.146	0.6	1.3	0.334	2.997	0.200
1	0.7	0.628	4.135	0.569	0.5	1.3	0.308	2.516	0.158
0.9	0.7	0.615	3.450	0.546	0.4	1.3	0.277	2.125	0.111
0.8	0.7	0.599	2.921	0.519	0.3	1.3	0.238	1.801	0.060
0.7	0.7	0.579	2.508	0.485	0.2	1.3	0.192	1.521	0.017
0.6	0.7	0.555	2.183	0.442					

**TABLE A2:** Model coefficients of correction factors $f_{d}$, $f_{d,\text{diss}}$, and $f_{p}$.

$a_{00}$	0.748395	$b_{00}$	0.087350	$c_{00}$	18.382846
$a_{01}$	0.647381	$b_{01}$	−1.852453	$c_{01}$	−25.746285
$a_{02}$	1.236903	$b_{02}$	−8.176982	$c_{02}$	15.179544
$a_{03}$	0.050597	$b_{03}$	2.930729	$c_{03}$	−2.935767
$a_{04}$	0.211290	$b_{04}$	0.050238	$c_{04}$	−8.651836
$a_{05}$	2.690815	$b_{05}$	−1.666054	$c_{05}$	26.896086
$a_{06}$	0.372779			$c_{06}$	0.822919
				$c_{07}$	0.131104
				$c_{08}$	0.579553

## References

[1] T. A. Osswald , J. P. Hernández‐Ortiz , Polymer Processing: Modeling and Simulation, Hanser Publishers, Munich 2006.

[2] Z. Tadmor , Z. G. Gogos , Principles of Polymer Processing, 2nd ed., Wiley & Sons Inc., Hoboken 2006.

[3] C. Hopmann , W. Michaeli , Extrusion Dies for Plastics and Rubber, 4th ed., Hanser Publishers, Munich 2016.

[4] J. F. Agassant , P. Avenas , P. J. Carreau , B. Vergnes , M. Vincent , Polymer Processing: Principles and Modelling, 2nd ed., Hanser Publishers, Munich 2017.

[5] M. J. J. Boussinesq , J. Math. Pures Appl 1868, 13, 377.

[6] Anonymous , Engineering 1922, 114, 606.

[7] H. S. Rowell , D. Finlayson , Engineering 1928, 126, 249.

[8] C. Maillefer . Ph.D. Thesis, University of Lausanne **1952** .

[9] S. Middleman , Trans Soc Rheol 1965, 9, 628.

[10] J. A. Wheesler , E. H. Wissler , AIChE J 1965, 11, 207.

[11] K. Palit , R. T. Fenner , AIChE J 1972, 9, 83.

[12] T. Sochi , Rheol. Acta 2015, 54, 745.

[13] C. Rauwendaal , Polymer Extrusion, 5th ed., Hanser Publishers, Munich 2014.

[14] G. Schenkel , Kunststoffe 1981, 71, 479.

[15] T. Köpplmayr , J. Miethlinger , Int. Polym. Process. 2013, 3, 322.

[16] J. L. White , D. Huang , Polym. Eng. Sci. 1981, 21, 1101.

[17] U. Lang , W. Michaeli , J. Vinyl Addit. Techn. 1998, 4, 65.

[18] Z. Rotem , R. Shinnar , Chem. Eng. Sci. 1961, 15, 130.

[19] M. Narkis , A. Ram , Polym. Eng. Sci. 1967, 7, 161.

[20] W. Roland , J. Miethlinger , Polym. Eng. Sci. 2018, 58, 2055.

[21] F. W. Kroesser , S. Middleman , Polym. Eng. Sci. 1965, 5, 230.

[22] P. J. Carreau . Ph.D. Thesis, University of Wisconsin‐Madison **1968** .

[23] K. Yasuda . Ph.D. Thesis, MIT, Cambridge **1968** .

[24] W. Roland , C. Marschik , M. Krieger , B. Loew‐Baselli , J. Miethlinger , J. Non‐Newton , Fluid Mech. 2019, 268, 12.

[25] S. Pachner , W. Roland , M. Aigner , C. Marschik , U. Stritzinger , J. Miethlinger , Int. Polym. Process. 2021, 4, 435.

[26] W. Roland , M. Kommenda , C. Marschik , J. Miethlinger , Polymers 2019, 11, 334. 30960318 10.3390/polym11020334 PMC6419227

[27] ANSYS Inc. , ANSYS Fluent, Release 18.2, Canonsburg 2017.

[28] C. Marschik , W. Roland , B. Loew‐Baselli , J. Miethlinger , J. Non‐Newton , Fluid Mech. 2017, 248, 27.

[29] S. Wagner , G. Kronberger , A. Beham , M. Kommenda , A. Scheibenpflug , E. Pitzer , S. Vonolefen , M. Kofler , S. Winkler , V. Dorfer , M. Affenzeller , in Advanced Methods and Applications in Computational Intelligence (Eds: R. Klempous , J. Nikodem , W. Jacak , Z. Chaczko ), Springer, Heidelberg 2014.

[30] W. Roland , C. Marschik , M. Kommenda , A. Haghofer , S. Dorl , S. Winkler , Int. Polym Process. 2021, 5, 529.

[31] C. Marschik , W. Roland , B. Loew‐Baselli , G. Steinbichler , SPE ANTEC Tech. Papers 2020.

[32] C. Marschik , W. Roland , M. Dörner , G. Steinbichler , V. Schöppner , Polymers 1919, 2021, 13. 10.3390/polym13121919 PMC8228144 34207753

[33] A. Hammer , W. Roland , C. Marschik , G. Steinbichler , J. Non‐Newton , Fluid Mech. 2021, 295, 618.

[34] U. Stritzinger , W. Roland , G. Berger‐Weber , G. Steinbichler , Polym. Eng. Sci. 2022, 62, 3721.

[35] S. J. Derezinski , J. Plast. Film Sheet. 1987, 3, 274.

[36] I. Sbarski , Int. Polym. Process. 1997, 4, 341.

[37] C. Marschik , M. Dörner , W. Roland , J. Miethlinger , V. Schöppner , G. Steinbichler , Polymers 2019, 11, 1488. 31547371 10.3390/polym11091488 PMC6780909

[38] C. Marschik , W. Roland , M. Dörner , G. Steinbichler , V. Schöppner , Polymers 1900, 2020, 12. 10.3390/polym13121919 PMC8228144 34207753

[39] W. Roland , C. Marschik , A. Hammer , G. Steinbichler , SPE ANTEC Tech. Papers 2020.

